# Five‐year outcomes of ADHD diagnosed in adulthood

**DOI:** 10.1111/sjop.12692

**Published:** 2020-11-20

**Authors:** Elin Nylander, Orestis Floros, Timea Sparding, Eleonore Rydén, Stefan Hansen, Mikael Landén

**Affiliations:** ^1^ Department of Psychology University of Gothenburg Gothenburg Sweden; ^2^ ADHD Unit Sankt Görans Hospital Stockholm Sweden; ^3^ Department of Clinical Neuroscience Karolinska Institutet Stockholm Sweden; ^4^ Department of Psychiatry and Neurochemistry Institute of Neuroscience and Physiology the Sahlgrenska Academy at University of Gothenburg Gothenburg Sweden; ^5^ Department of Medical Epidemiology and Biostatics Karolinska Institutet Stockholm Sweden

**Keywords:** Adult, attention‐deficit/hyperactivity disorder, follow‐up studies, prognosis, treatment outcome

## Abstract

There is a dearth of long‐term follow‐up studies of adults diagnosed with ADHD. Here, the aim was to evaluate long‐term outcomes in a group of ADHD patients diagnosed in adulthood and receiving routine psychiatric health care. Adults diagnosed with any type of ADHD (*n* = 52) and healthy controls (*n* = 73) were assessed at baseline and at a 5‐year follow‐up, using Global Assessment of Functioning (GAF), Clinical Global Impression (CGI), Brown ADD Scale (BADDS) and Adult ADHD Self‐Report Scale (ASRS). A multivariate regression method was used to identify factors predicting 5‐year outcomes, including baseline ratings, medication intensity, comorbidity, intelligence quotient (IQ), age, and sex. After 5 years, ADHD patients reported fewer and/or less severe symptoms compared to baseline, but remained at clinically significant symptom levels and with functional deficits. Baseline self‐reports of ADHD symptoms predicted their own 5‐year outcome and low baseline functioning level predicted improved global functioning at follow‐up. Factors previously reported to predict short‐term outcomes (i.e., medication, comorbidity, IQ, age, and sex) did not anticipate long‐term outcomes in present study.

## INTRODUCTION

It was long assumed that Attention‐Deficit/Hyperactivity disorder (ADHD) affected children only. But even though ADHD symptoms decline by age (Faraone, Biederman & Mick, 2006; Weiss, Murray & Weiss, 2002), most ADHD patients (75–90%) have lingering symptoms with at least some functional deficit as adults (Biederman, Petty, Clarke, Lomedico & Faraone, 2011; Ingram, Hechtman & Morgenstern, 1999; Kessler, Adler, Barkley *et al*., 2005; Sibley, Mitchell & Becker, 2016). Because of this, awareness of ADHD in adults has rapidly increased (Asherson, Buitelaar, Faraone & Rohde, 2016). The estimated prevalence of adult ADHD is 2.8–3.4% (Fayyad, De Graaf, Kessler *et al*., 2007, Fayyad, Sampson, Hwang *et al*., 2017). In a follow‐up study of the Multimodal Treatment of Attention‐Deficit/Hyperactivity Disorder (MTA)‐sample (N = 453) as 25 year olds, initial ADHD symptom severity, parental mental health, and childhood comorbidity, did all predict persistent ADHD symptoms in adulthood (Roy, Hechtman, Arnold *et al*., 2016).

Adult ADHD is associated with poor functioning in everyday life (Asherson *et al*., 2016). For example, a Swedish study of several thousand adult ADHD patients showed that only around one third were employed, that the mean income was substantially reduced compared to the general population, and that a whopping 64% of the men had been convicted of crime (Chang, Lichtenstein, D’Onofrio, Sjölander & Larsson, 2014). Similarly bleak functional outcomes in adult ADHD have been reported in other studies (reviewed by Hervey, Epstein & Curry, 2004). Adult ADHD is also commonly associated with other psychiatric disorders (Giacobini, Medin, Ahnemark, Russo & Carlqvist, 2018), such as anxiety, depression, or substance abuse (Chen, Hartman, Haavik *et al*., 2018).

In children and adolescents, studies have shown clear short‐term beneficial effects of psychostimulant medication but its long‐term consequences are less clear. For example, in the MTA‐study, treatments including psychostimulant medication were superior to non‐pharmacological treatment options at a 14‐month follow‐up but these beneficial effects were not discernible one year later (reviewed by Hinshaw, Arnold & MTA Cooperative Group, 2015).

For adult ADHD, psychostimulant drugs are equally regarded as first‐line treatments, at least in the short run, and as part of a multimodal approach including viz. psychoeducation (Kooij, Bijlenga Salerno *et al*., 2019). However, they appear less efficacious and less well tolerated in adults than in children/adolescents (Cortese, Adamo, Del Giovane *et al*., 2018). Medication in adults has been shown to confer several beneficial effects of functioning, beyond alleviating symptoms, including reducing the rate of serious traffic accident and criminal behavior (Chang *et al*., 2014; Lichtenstein, Halldner, Zetterqvist *et al*., 2012). However, discontinuation or stop/start patterns of ADHD medication are common. For example, Bejerot, Rydén and Arlinde (2010) found that only 50% of adult ADHD patients remained on medication 2 years after commencement. On the other hand, adult ADHD patients can be made to adhere more closely to stimulant treatment during more than 3 years by close monitoring and encouragement (Torgersen, Gjervan, Nordahl & Rasmussen, 2012).

Relatively little is known about the long‐term (≥ 2 years) trajectory regarding symptoms and functioning levels of adults diagnosed with ADHD as adults (Harpin, Mazzone, Raynaud, Kahle & Hodgkins, 2016; Shaw, Hodgkins, Caci *et al*., 2012). However, we first know that most patients remain impaired and that multiple symptoms persist. For example, a 7‐year study by Karam, Breda, Picon *et al*. (2015) reported from a sample of adult ADHD patients diagnosed in childhood, that only 12.4 % of the patients (mean age 34.1; 49.9% males) were remitted (less than 4 ADHD criteria), even though about one third (30.2%) of the total sample met fewer ADHD criteria at follow‐up than at baseline. Second, high rates of baseline ADHD symptoms, comorbidity (i.e., oppositional defiant disorder and social phobia) and parental psychopathology are all associated with persistent ADHD in adult age (Biederman *et al*., 2011; Karam *et al*., 2015; Lara, Fayyad, de Graaf *et al*., 2009; Lensing, Zeiner, Sandvik & Opjordsmoen, 2013). Third, while treatment responders tend to stay on medication, difficulties with compliance (‘treatment responders’ defined according to self‐reported symptoms in Bejerot *et al*., 2010; Lensing *et al*., 2013) and dropouts (Bejerot *et al*., 2010; Bijlenga, Kulcu, van Gellecum, Eryigit & Kooij, 2017; Edvinsson & Ekselius, 2017; Soendergaard, Thomsen, Pedersen *et al*., 2016) are common in this group.

There are but a few naturalistic studies tracking the long‐term clinical and functional outcomes in adult ADHD (Hodgkins *et al*., 2012), calling for more studies to understand long‐term trajectory regarding symptoms and functioning levels in ADHD as it presents in real‐world settings (Cortese *et al*., 2018; Shaw *et al*., 2012). Intensified focus on the long‐term consequences of ADHD and its treatment is important because of the heightened awareness among laypeople, high heritability (74%, because of the tendency of ADHD to run in families and through generations; Faraone & Larsson, 2019) and the profound effects ADHD has on life quality (see for example Chang *et al*., 2014; Dalsgaard, Østergaard, Leckman, Mortensen & Pedersen, 2015; Sun, Kuja‐Halkola, Faraone *et* *al*., 2019).

### Aims

In the present study, we followed 52 persons diagnosed with ADHD in adulthood over 5 years. Our aim was to evaluate long‐term outcomes in a group of carefully diagnosed ADHD patients, since ADHD is a life‐long impairment associated with poor functioning in daily life. We compared self‐report symptom ratings and clinicians’ ratings of symptom severity at baseline and at the 5‐year follow‐up. Employing multivariate regression methods, we attempted to identify outcome (symptom severity and real‐life functioning) predictors using rating scores at baseline along with measures of medication intensity, psychiatric comorbidity, cognitive ability, age, and sex.

## METHODS

### Patients

The present study sample is part of a project within the Northern Stockholm Mental Health Service, the St Göran project, which assesses patients with ADHD (and bipolar disorder, see Pålsson, Sellgren, Rydén *et al*., 2017) over several years. Patients with ADHD were enrolled from a tertiary outpatient clinic specialized in assessment and treatment of ADHD. Experienced board‐certified psychiatrists (ER or OF) conducted structured anamnestic interviews with the patients. The interview structure relies upon the clinical assessment instrument Affective Disorder Evaluation (ADE) with its origin in a bipolar disorder study (Sachs, Thase, Otto *et al*., 2003). For the purpose of diagnosing ADHD in the project, the protocol was complemented with a section covering the DSM‐IV diagnostic criteria for ADHD (American Psychiatric Association, 2000). The ADE also includes a social anamnesis, medical history, and family history. In addition, the MINI International Neuropsychiatric Interview (MINI; Sheehan, Lecrubier, Sheehan *et al*., 1998) was used to screen for psychiatric diagnoses other than ADHD and bipolar disorder, which are covered in the ADE. The Wender Utah Rating Scale (WURS; Ward, Wender & Reimherr, 1993) was used to assess childhood ADHD symptoms. The Adult ADHD Self‐report Scale (ASRS; Kessler *et al*., 2005) and the Brown Attention‐Deficit Disorder Scales (BADDS; Brown ADD Scales; Rucklidge & Tannock, 2002) were used to assess current ADHD symptoms. Clinicians used the Global Assessment of Functioning (GAF) and Clinical Global Impression‐Severity (CGI‐S) to rate the patients functioning and symptoms, respectively. All available sources of information, encompassing patient interview, case records and, if available, interview with next of kin were utilized in the diagnostic assessment.

Present data were extracted from the St. Göran research database, a Structured Query Language (SQL) based database hosted by the University of Gothenburg, in January 2016. By that time it contained 91 patients with ADHD and 116 controls, all recruited in Stockholm. In this study, we only included patients with complete or near‐complete data from two time‐points (psychiatric interview and self‐reports at baseline and 5‐year follow‐up). This left 52 patients with ADHD and 73 controls for participation in present study. For background characteristics see Table 1 in the Results section.

**Table 1:** Demographics and test scores at baseline for patients and healthy controls

	Patients *n* = 52		Healthy controls *n* = 73		
	*Average*	*Spread*	*Average*		*Spread*
Age	35^a^	16^b^		37^a^	19^b^
WURS ADHD	50.3^c^	45‐55.7^d^		9.5^c^	8‐11^d^
WAIS‐III FSIQ	114^d^	108‐119^d^		116^d^	113‐118^d^
Sick leave, days	2^a^	15^b^		0^a^	4^b^

Abbreviations: WURS, Wender Utah Rating Scale; ADHD, Attention‐deficit/Hyperactivity Disorder; WAIS, Wechsler Adult Intelligence Scale; *Notes*: FSIQ, Full Scale Intelligence Quotient.

^a^ Median;

^b^ Interquartile range;

^c^ Mean;

^d^ 95% CI;

^e^ n = 26 (50%);

^f^ n = 67 (89%).

The control group consisted of population‐based controls that were randomly selected through Statistics Sweden (SCB) and contacted by mail. A research nurse contacted individuals who volunteered to participate in the study. Controls were scheduled for a one‐day comprehensive assessment comprising a psychiatric interview by experienced clinicians using selected parts of the ADE and the MINI to exclude psychiatric disorders. Control persons were screened for substance abuse in several ways: during the telephone interview, during the psychiatric in‐person interview, through the self‐report Alcohol Use Disorders Identification Test (AUDIT) and Drug Use Disorders Identification Test (DUDIT), and also by determining serum concentrations of carbohydrate‐deficient transferrin (CDT). Overconsumption of alcohol or other drug abuse led to exclusion. Other exclusion criteria were neurological conditions (apart from mild migraines), untreated endocrinological disorders, pregnancy, dementia, recurrent depressive disorder, personality disorders (based on the psychiatric interview and assessment with the Structured Clinical Interview for DSM‐IV Axis II Personality Disorders, screen questionnaire (SCID‐II‐SQ), and a family history of schizophrenia or bipolar disorder in first‐degree relatives.

The attrition was 39 patients, whereof nine lacked self‐report data at baseline. For the remaining 30 patients lost to follow‐up, baseline scores were compared with those who were re‐evaluated at follow‐up (the study sample). The baseline averages did not differ between the study sample and those lost to follow‐up: BADDS *M* = 65.9; *SD* = 23.3 (attrition) vs. *M* = 63.1; *SD* = 20.2 (study sample); ASRS *M* = 42.9; *SD* = 9.7 (attrition) vs. *M* = 41.0; *SD* = 10.4 (study sample); GAF *M* = 64.5; *SD* = 8.6 (attrition) vs. *M* = 65.8; *SD* = 9.7 (study sample); CGI‐S *M* = 4.0; *SD* = 0.6 (attrition) vs. *M* = 3.8; *SD* = 0.7 (study sample). None of these differences were statistically significant by the t‐test (*t'*s = 0.57– 1.32 and all *p*‐values> 0.05).

### Psychometric instruments

*Brown ADD scale (BADDS)* is a 40‐item self‐report scale that assesses executive functioning. Individual items are rated on a scale from 0 to 3 (never to almost daily). The items are clustered into five subscales. BADDS is primarily designed to measure the inattentive part of the ADHD symptomatology. Total score can range from 0 to 120. The clinical cutoff score 50 indicates ‘probable ADHD’ (Brown *et al*., 2011).

*The WHO Adult ADHD Self‐Report Scale (ASRS)* has 18 items, which correspond to the 18 diagnostic criteria of ADHD symptoms in the diagnostic manual DSM 5 (American Psychiatric Association, 2013), and includes questions about both the inattentive and the hyperactive/impulsive symptoms. The responses are given in a five‐point Likert‐scale from 1 (never) to 5 (always). The ASRS has shown good reliability and validity for evaluation of ADHD in adults (Adler, Spencer Faraone *et al*., 2006). The clinical cutoff score of 24 (for either inattention or hyperactivity/impulsivity) indicating ‘highly likely ADHD’; 17–23 point indicating ‘likely ADHD’ and 0–16 indicating ‘unlikely ADHD’ for this full version (ASRS‐18) as proposed by Yeh, Gau, Kessler, and Wu (2008) was adopted in present study.

*Global Assessment of Functioning (GAF; GAF functioning and GAF Symptom)* ranges from 100 (extremely well‐functioning/ no symptoms) to 1 (severely impaired/ severe psychiatric symptoms) and is used to rate overall psychological functioning plus social and occupational functioning (how well the patient is handling various everyday problems) and psychiatric symptoms. The GAF has some reliability and validity issues but is widely used in routine clinical settings (Monrad‐Aas, 2010; Piersma & Boes, 1997; Söderberg, Tungström & Armelius, 2005).

*Clinical Global Impression Scale – symptom severity (CGI‐S)* is a 3‐items scale measuring symptom severity, global improvement and therapeutic response. In the present study, the symptom severity item was included, which summarizes the clinician’s global impression of symptom severity. The CGI‐S is rated on a 7‐point scale, from 1 (not ill at all) to 7 (extremely ill).

*Wender Utah ADHD Rating Scale (WURS)* is a 61‐item retrospective self‐report scale, based on DSM‐criteria, used to estimate childhood ADHD symptoms in adults (Ward *et al*., 1993). Twenty‐five questions are directly related to ADHD and add up to a summary ADHD score, which was used in present study. The participant recalls symptoms from his/her childhood and responds on a five‐point Likert scale. The Swedish version of WURS self‐report has good psychometric properties (Kouros, Hörberg, Ekselius & Ramklint, 2018).

### Statistics

Descriptive statistics are presented as means/medians and 95% confidence intervals/interquartile ranges, unless noted otherwise. To evaluate temporal patterns and treatment effects a series of paired t‐tests (IBM SPSS Statistics for Mac, Version 22.0. Armonk, NY) were conducted; statistical significance for BADDS total and its five subscales was adjusted according the sequential Bonferroni‐Holm method to avoid Type I errors (see Holm, 1979). Effect sizes are expressed as partial eta‐squared (*η^2^*); the computation of *η^2^* following pairwise t‐tests employed an online calculator (http://www.psychometrica.de/effect_size.html). The definitions of *η^2^* magnitude are 0.01 (small), 0.06 (medium), and 0.14 (large) according to Cohen (1988).

We used a multivariate regression technique, Orthogonal Partial Least Squares (OPLS; Eriksson, Byrne, Johansson, Trygg & Vikström, 2013), in order to identify factors at baseline predicting outcomes 5 years later. The OPLS regression procedure (SIMCA‐P 13.0, Sartorius Stedim Biotech, Göttingham, Germany) forms a latent component composed of that portion of the systematic variation in the predictor set (i.e., baseline data in the present case) that is specifically related to the variation in the outcome variable (i.e., follow‐up data in the present case). It does this by leaving out the systematic variation among the predictors that is uncorrelated (i.e., orthogonal) to it. In this way, the OPLS regression procedure filters away irrelevant information in the predictor data set and maximizes the explained covariance between predictors and outcome (Eriksson *et al*., 2013).

By default the SIMCA software transforms the data by unit variance scaling and mean centering. Similarly, the solidity of the predictive component is determined according to the software’s default cross‐validation significance test, in which all data are left out once in a seven leave‐out series. In this way, a number of parallel models are developed; if they are sufficiently similar the model is deemed significant (Eriksson *et al*., 2013).

The relationship between the dependent variable and the predictive component is described by a number of parameters, such as each predictor’s scaled and centered regression coefficient. The Variable Influence on Projection (VIP) summarizes the importance of the various independent variables for the predictive component. Variables with VIPs ≥ 1 are considered very significant and important for the model (Eriksson *et al*., 2013), and accordingly this VIP‐criterion was used for interpreting the present OPLS results. In addition, there are two important measures describing the quality of a particular OPLS model: *R^2^X* is the fraction of the variation of the predictors modeled by the component; *R^2^* is the fraction of the variation in the dependent variable modeled by the predictors.

In comparison to regular multiple linear regression, OPLS deals well with collinearity and missing data. Importantly, it (and related techniques) was developed to handle data sets with many variables relative to the number of observations/participants (‘short‐and‐wide’ matrices) and it is also robust to noise in both the predictor‐ and the dependent datasets (Eriksson *et al*., 2013). Accordingly, OPLS was considered well suited for the present type of clinical data with a large number of inter‐correlated variables with relatively few participants. OPLS models get more robust when predictors overlap (Eriksson *et al*., 2013), which is why we do not discard any of the intersecting symptom rating scales employed in the present research.

### Ethics approval and consent to participate

The Regional Ethics Committee in Stockholm approved this study (2005/554‐31/3), which was conducted in accordance with the latest Helsinki Protocol. All patients and controls consented both orally and in writing to participation in this study.

## RESULTS

### Background characteristics

Table 1 presents background information on the participants. Fifty‐two adults diagnosed with ADHD were included (21 females; 40.4% and 31 males; 59.6%). At baseline, 19 (37%) patients had comorbid depression or anxiety disorder, three (6%) had comorbid developmental disorder (i.e., autism spectrum disorder), and two (3%) patients a comorbid personality disorder. No patients reported symptoms of present substance abuse. Thirty‐one patients (60%) scored 46 or higher in WURS rating scale, indicating recalled impairing symptoms of childhood ADHD.

As our study was a routine clinical practice observational study, we had no control over medication type, discontinuation, doses, or visits over the course of 5 years. However, according to the medical records, 44 (84.6%) of the patients had medicated with central stimulants during at least one prescription period. Thirty‐four (65.4%) were on medication at both baseline and follow‐up. Although we recognize that this does not necessarily imply continuous medication, we nevertheless assumed that this group were on ADHD medication more regularly than the rest of the patients. They therefore formed the ‘medicated’ group in the statistical analyses. However, for 22 in this group of 34, the exact number of months being on medication was available: the median was 48 months but with considerable variability (interquartile range: 26 months). For the remaining patients, forming the ‘non‐medicated’ group, eight (15.4%) did not use medication at any of the two time points, eight (15.4%) medicated at baseline but not at follow‐up, while two (3.8%) medicated only at follow‐up.

There were no apparent relationships between the length of medical treatment and outcomes according to the GAF‐ and BADDS within the group of 22 patients for which the exact number of being on medication was known (*r*’s −0.19 and 0.15, respectively). A power estimation indicate that a group size of 40 to 60 would have been required to ascertain statistical significance of an association of this size at α of 0.05 and β of 0.80.

There were significant relationships between self‐rated ADHD‐symptoms (BADDS and ASRS) on the one hand and clinician‐rated functioning level (GAF functioning) on the other, at both baseline and follow‐up. The Spearman *r*s were: baseline BADDS‐GAF‐F: *r *= −0.316, *p* = 0.024; 5 year follow‐up BADDS‐GAF‐F: *r* = −0.434, *p* = 0.001; 5 year follow‐up ASRS‐GAF‐F: *r* = −0.325, *p* = 0.019); the exception was the non‐significant ASRS‐GAF‐F correlation at baseline: *r* = 0.011, *p* = 0.936.

For comparative purposes a group of 73 healthy controls were included (see Table 1).

### Change in ADHD symptoms over time

**Fig. 1:**
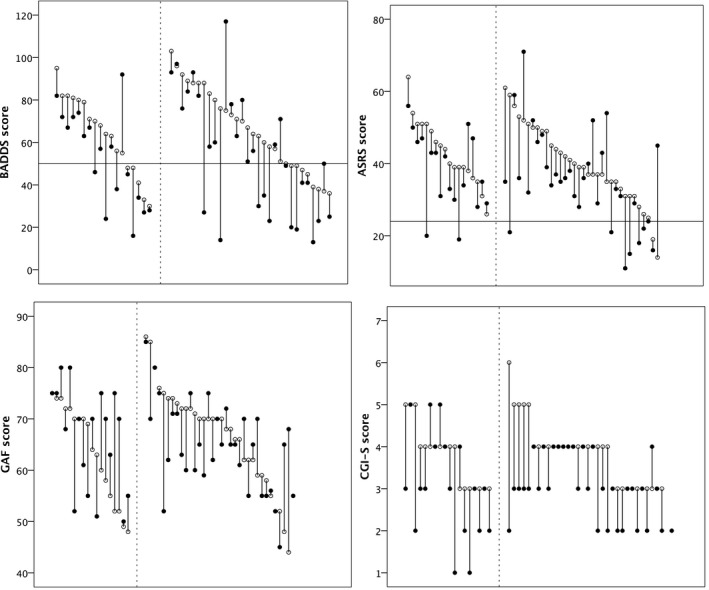
Drop‐line plots showing individual scores on BADDS, ASRS, CGI‐S and GAF at baseline (open circles) and at the 5‐year follow‐up (filled circles) in adult patients with ADHD. The horizontal lines denote clinical cut‐offs. Participants to the right of the vertical dotted line are patients receiving ADHD medication at both baseline and follow‐up; those to the left represent the rest.

**Fig. 2:**
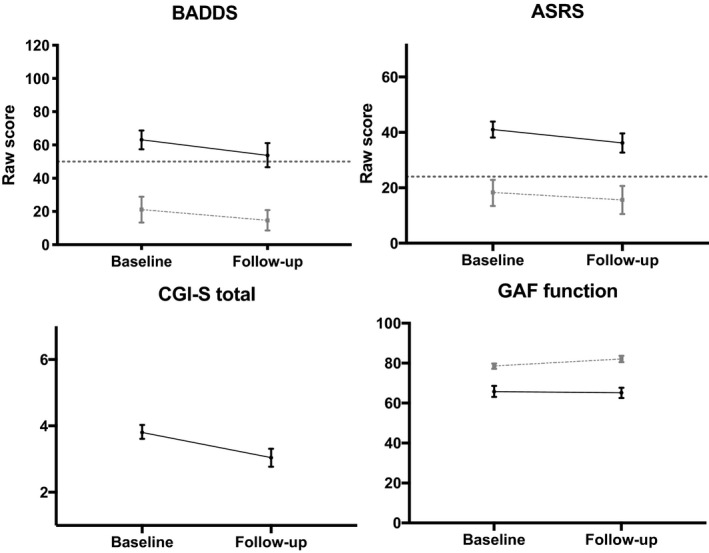
Self‐reported symptomatic changes, functional changes, and changes in clinicians’ ratings of symptoms, from baseline to 5‐year follow‐up. Black solid lines represent the patients; faded, dashed lines represent controls, and the dotted line marks clinical cut off. Means (M) and 95% Confidence Intervals (CIs) are presented. Note that the BADDS values for healthy controls are based on 10 respondents only.

Figure 1 shows each individual patient’s baseline and follow‐up scores over the course of five years. Figure 2 summarizes these data along with data from the healthy controls. The difference between the scores within the ADHD group was assessed using paired *t*‐tests (see Table 2 for statistical details). As to the BADDS, the patients’ total score was significantly lower at follow‐up (*p* = .001), but the effect size was small (*η^2^* = 0.05) and the average patient scored above the clinical cut‐off for BADDS also at follow‐up. Among the constituent BADDS subscales, Activation (*p* = 0.006, *η^2^* = .04), Attention (*p* = 0.000, *η^2^* = 0.06), and Effort (*p* = 0.001, *η^2^* = 0.05) were significantly improved at follow‐up compared with baseline (Table 2). Scores on the remaining subscales remained unchanged (Table 2).

**Table 2:** Measures at baseline and follow‐up for self‐reported BADDS and ASRS along with clinicians’ ratings of CGI‐S‐S and GAF symptoms and GAF functions. Means (M) and 95% CIs are presented

	Baseline		Follow‐up					
	*M*	95 % *CI*	*M*	95 % *CI*	*df*	*t*	*p*	*η^2^*
BADDS Total	63.1	57.4‐68.8	53.8	46.6‐61.1	50	3.71	.001^a^	.05
BADDS Activation	16.2	14.7‐17.7	14	12.2‐15.8	50	2.89	.006^a^	.04
BADDS Attention	15.6	14.1‐17.1	12.7	10.7‐14.6	50	3.96	.000^a^	.06
BADDS Effort	13.7	12.1‐15.2	11.2	9.4‐13.1	50	3.71	.001^a^	.05
BADDS Affect	9.1	8‐10.2	8.3	6.9‐9.6	50	1.58	.120^a^	.01
BADDS Memory	8.5	7.4‐9.7	7.7	6.4‐9	50	1.87	.067^a^	.01
ASRS	41.0	38.1‐43.9	36.2	32.7‐39.6	51	2.88	.006	.04
CGI‐S	3.8	3.6‐4	3	2.8‐3.3	49	4.98	.000	.16
GAF Function	65.8	63.1‐68.6	65.2	62.6‐67.7	50	.34	.738	.00
GAF Symptom	66.9	65‐68.8	64.9	62.5‐67.3	45	1.35	.185	.02

Notes

BADDS, Brown ADD Rating Scale; ASRS, The WHO Adult ADHD Self‐report Scale; CGI‐S, Clinical Global Impression‐Symptoms; GAF, Global Assessment of Functioning.

^a^ Adjusted according the sequential Bonferroni‐Holm method.

The patients also reported clinical ADHD symptom levels on the ASRS at both time points, but the ASRS scores were lower (i.e., improved) at follow‐up compared with baseline (*p* = 0.006, *η^2^* = 0.04). As to the CGI‐S scale, running from 1 (healthy) to 7 (extremely ill), the patients significantly improved over time (*p* = 0.000), and the effect size was higher (medium) than for the other scales (*η^2^* = 0.16). Concerning the GAF functioning/symptom scales, the patients’ baseline‐ and follow‐up scores did not differ statistically (Table 2).

Controls showed a significant improvement in the ASRS self‐report [*t*(61)*=* 3.18, *p =*0.002, *η^2^* = 0.05] and in the GAF functioning scores [*t(69) =* −3.68*, p =*0.000, *η^2^* = 0.08] from baseline (ASRS: M = 19.5, 95% CI = 16.4‐22.7; GAF functioning: M = 78.5, 95% CI = 77.2‐79.9) to follow‐up (ASRS: M = 14.4, 95% CI = 12.3–16.4; GAF functioning: M = 82.2, 95% CI = 80.6–83.7). No statistical test was run for the BADDS in controls because only 10 out of 73 controls completed the assessment at both time points.

As seen in Fig. 1, quite a few patients had CGI‐S scores ≤ 3, indicating that they were judged to be only mildly affected by the disorder. We analyzed treatment effect separately within the mildly and severely affected group, but we did not detect any differences (data not shown).

### Baseline scores in relation to outcome

To test if characteristics at baseline predict follow‐up scores, the scale scores at baseline, plus sex, age, comorbidity (0, 1), and full scale IQ (WAIS‐III) at baseline were used as predictors in a series of OPLS models. ADHD medication was coded as 1 for patients receiving medication at *both* baseline and follow‐up, and as 0 for the rest. In all, 17 predictors were used to model BADDS total score, ASRS total score, CGI‐S score, and GAF function score at the 5‐year follow‐up. Table 3 shows a correlation matrix of the variables included in the modeling.

**Table 3:** Spearman correlation matrix for the variables included in the OPLS models

	BADDS Total	ASRS Total	WURS ADHD	GAF Symptom	GAF Function	CGI‐S	WAIS‐III FSIQ	Sex	Age	Comorbidity	Sickleave	Medication
BADDS Total												
ASRS Total	0.515**											
WURS ADHD	0.047	0.017										
GAF Symptom	‐0.435**	−0.204	0.140									
GAF Function	−0.287*	−0.027	0.129	0.707**								
CGI−S	0.169	−0.159	0.121	−0.306*	−0.394**							
WAIS‐III FSIQ	0.215	0.009	−0.565**	−0.169	−0.001	−0.081						
Female	−0.104	−0.224	−0.046	0.229	0.226	−0.199	0.324					
Age	0.096	0.109	−0.049	−0.364**	−0.331*	−0.129	0.005	0.128				
Comorbidity	0.059	−0.114	−0.022	−0.180	−0.176	0.481**	−0.054	−0.201	0.048			
Sick leave	0.139	−0.094	0.090	−0.354*	−0.582**	0.580**	−0.041	−0.252	0.107	0.304		
Medication	0.022	0.156	−0.394**	−0.105	−0.164	−0.043	0.070	−0.143	0.143	0.225	−0.024	
BADDS Total 5y	0.661**	0.272	0.170	−0.337*	−0.235	0.035	−0.053	0.039	0.224	0.086	−0.014	−0.011
ASRS 5y	0.523**	0.452**	0.170	−0.242	−0.061	−0.010	0.044	−0.004	0.205	−0.002	−0.139	0.108
CGI−S 5y	0.358*	0.121	0.099	−0.461**	−0.485**	0.215	0.155	−0.094	0.238	0.175	0.137	0.013
GAF Function 5y	−0.233	−0.037	−0.119	0.274	0.435**	−0.093	0.027	0.050	−0.076	−0.089	−0.126	0.098

Notes

**Correlation is significant at the 0.01‐level (2‐tailed); * Correlation is significant at the 0.05‐level (2‐tailed).

BADDS, Brown ADD Rating Scale; ASRS, The WHO Adult ADHD Self‐report Scale; CGI‐S, Clinical Global Impression‐Symptoms; GAF, Global Assessment of Functioning; WAIS, Wechsler Adult Intelligence Scale; FSIQ, Full Scale Intelligence Quotient.

The resulting model for the self‐report BADDS at follow‐up, significant by cross‐validation, used approximately 22% of the variation in the predictor set to form a component explaining 51% of the variation in the BADDS total scores at follow‐up. Table 4 shows that high BADDS scores were associated with worse outcome in BADDS at follow‐up. No other predictor had VIPs on or above threshold. Similarly, baseline BADDS also predicted ASRS self‐report scores along with ASRS baseline scores (data not shown).

**Table 4:** Regression coefficients and VIP’s derived from OPLS modeling of 5‐year outcomes

	BADDS at 5 years *R^2^X* = 0.22 *R* ^2^ = 0.51		CGI‐S at 5 years *R^2^X* = 0.19 *R^2^* = 0.35		GAF function *Improvement score* *R^2^X* = 0.14 *R^2^* = 0.44	
Predictor	Regression coefficient^a^	VIP^b^	Regression coefficient^a^	VIP^b^	Regression coefficient^a^	VIP^b^
BADDS Total	0.15	**1.79**	0.05	**1.41**	−0.03	**1.02**
BADDS Effort	0.17	**1.56**	0.10	**1.27**	−0.04	0.89
BADDS Attention	0.12	**1.49**	0.05	**1.05**	−0.13	**1.01**
BADDS Activation	0.07	**1.49**	0.04	**1.25**	−0.02	0.88
BADDS Memory	0.13	**1.47**	0.00	**1.06**	0.04	0.68
BADDS Affect	0.13	**1.42**	−0.01	**1.29**	0.07	**1.08**
ASRS Total	−0.02	1.06	−0.03	0.94	−0.04	0.82
WURS ADHD	0.12	0.55	0.08	0.16	−0.13	0.76
GAF Symptom	−0.07	0.97	−0.18	**1.64**	−0.14	**1.55**
GAF Function	−0.04	0.68	−0.22	**1.63**	−0.22	**1.85**
CGI‐S‐S	−0.05	0.31	0.07	**1.13**	0.09	**1.01**
WAIS‐III FSIQ	−0.05	0.25	0.05	0.36	0.00	0.10
Female	−0.09	0.89	−0.01	0.48	0.05	0.81
Age	0.10	0.59	0.11	0.80	0.10	0.85
Comorbidity	0.03	0.17	0.06	0.87	0.01	0.78
Sick leave	−0.05	0.36	0.01	0.91	0.17	1.32
Medication	0.03	0.53	0.03	0.31	−0.08	0.87

VIP, Variable Influence on Projection; BADDS, Brown ADD Rating Scale, ASRS, The WHO Adult ADHD Self‐report Scale; WURS, Wender Utah Rating Scale, GAF, Global Assessment of Functioning; CGI‐S, Clinical Global Impression‐Symptoms, WAIS, Wechsler Adult Intelligence Scale; FSIQ, Full Scale Intelligence Quotient.

^a^ Centered and scaled.

^b^ Values ≥ 1 are important for the model.

As to the clinicians’ CGI‐S ratings at follow‐up, approximately 19% of the variation in the predictor set related to 35% of the outcome. In this case, the two GAF scales (functioning and symptoms) constituted the strongest predictors (Table 4). Clinicians’ ratings on the GAF functioning scale at follow‐up could not be related to the predictor set (data not shown).

**Fig. 3:**
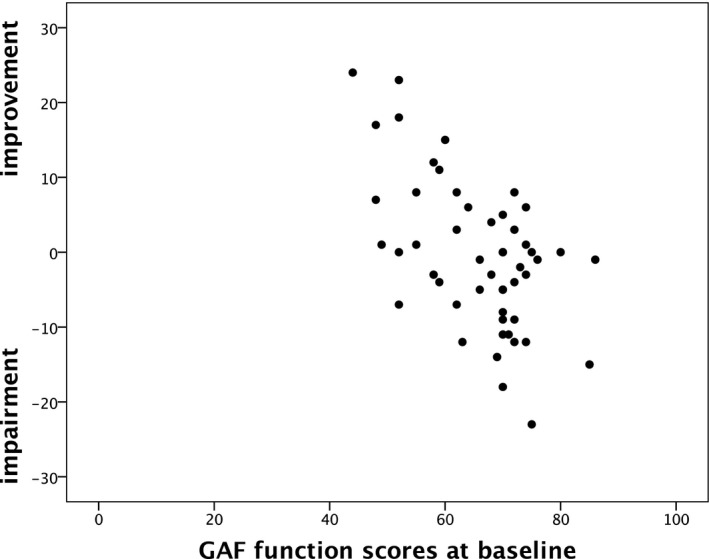
Scatterplot showing the relationship between GAF function scores at baseline and improvement‐/impairment over 5 years in adult ADHD patients. Improvement/impairment scores were computed by subtracting GAF follow‐up scores from GAF baseline scores

In a complementary approach, we investigated predictors of improvement over the course of 5 years. Alas, as to the BADDS self‐report scales and the CGI‐S, the attempts were unsuccessful, in the former case because of non‐significance of the model, and in the latter because of limited variation in the baseline/follow‐up scores. However, with regard to the GAF functioning scale, OPLS used 14% of the predictive variance to explain 44% of the GAF functioning improvement scores (Table 4). The GAF functioning baseline score was the strongest predictor for improvement in GAF functioning score, followed by GAF symptom and the number of sick leave days. Figure 3 illustrates the relationship between baseline GAF function scores and improvement/ impairment over 5 years: lower (worse) GAF functioning scores were associated with a larger improvement whilst the reverse was true for patients with higher (better) GAF scores (Spearman *r =* **−**0.47).

## DISCUSSION

We followed 52 individuals diagnosed with ADHD in adulthood over 5 years using clinical interviews, self‐reports (BADDS, ASRS, WURS), and clinical ratings (CGI‐S, GAF). The main findings were that the ADHD symptom burden decreased over the course of 5 years according to both self‐reports and clinicians’ judgements. ADHD symptom rating scales (ASRS and BADDS) predicted their own 5‐year outcome, such that high scores at baseline predicted worse outcome. Lower (worse) clinician‐rated functioning scores (GAF) were associated with a larger improvement. Retrospective WURS childhood ADHD ratings, comorbidity and medical treatment had no bearing on the 5‐year outcomes.

With respect to patients’ self‐reports, the decrease over the course of 5 years was significant in the statistical sense, but the averages remained at clinical levels and the effect sizes were weak. Clinicians’ ratings (CGI‐S) indicated a more robust improvement with a higher effect size. The discrepancy between clinicians’ and patients’ reports provides yet another example of partial patient‐informant disconcordance (De Los Reyes, Augenstein, Wang *et al*., 2015). Previous studies have noted that patients with ADHD tend to underestimate their symptoms in comparison with other informants (Swanson, Arnold, Molina *et al*., 2017). The modest but significant decrease in ADHD symptoms documented here might reflect a true symptom reduction and/or an increased ability to cope with the difficulties, making the symptoms and functional deficits less obvious and impairing; indeed, symptom severity and functional deficits were inversely related to one another in the present study. Alternatively, the decrease might be due to a replacement of more overt symptoms as hyperactivity and impulsivity by more subtle symptoms like mental restlessness and excessive mind wandering as described by Kooij *et al*. (2019). Such inner symptoms of adult ADHD may be insufficiently covered in the instruments used in the present study. Finally, milder ADHD symptoms at follow‐up compared with baseline could also simply be due to regression‐to‐the‐mean phenomena. In line with our findings, age‐dependent declines in ADHD symptoms have been demonstrated in earlier studies (Biederman, Mick & Faraone, 2000; Faraone *et al*., 2006; Srebnicki, Kolakowski & Wolanczyk, 2013). Importantly, however, symptoms at follow‐up remained at clinical levels despite improvement over 5 years, confirming the well‐known persistence of ADHD symptoms (Roy *et al*., 2016; Sibley *et al*., 2016).

Interestingly, self‐rated ADHD symptoms (ASRS scores) and clinician‐rated functional impairment (GAF scores) improved also in the healthy controls during the study period, despite ASRS being subclinical at baseline and the controls’ GAF functioning level being high (in the span 71–80). Speculatively, and among several possibilities, these changes might be reflections of the positive personality development documented in adult healthy people, involving higher levels of conscientiousness and lower levels of neuroticism on average (reviewed by Roberts & Mroczek, 2008). Thus, as adults mature they tend to get better at impulse control/delayed gratification (increased conscientiousness) and to become more emotionally stable (less neurotic), changes that would be expected to be beneficial when dealing with everyday hassles and work demands. In this context it is interesting to note that the personality of adults with ADHD is characterized by low levels of conscientiousness (hard‐working/control impulses/delay gratification while working towards goals) and high levels of neuroticism (Nigg, John, Blaskey *et al*., 2002; Parker, Majeski & Collin, 2004).

The regression models showed that the baseline, self‐rated ADHD symptom scale (BADDS scores) had broader predictive value than the other self‐rated ADHD symptom scale (ASRS;Table 4). Thus, baseline BADDS scores not only predicted its own score 5 years later, but were also relevant for the understanding of the follow‐up clinician‐rated symptomatic (CGI‐S) and functional impairment (GAF‐F) ratings. A similar pattern emerges when one studies the correlation matrix presented in Table 3: BADDS correlate with multiple variables, including ASRS at baseline, whereas ASRS only correlates with itself 5 years later and with BADDS at baseline. This differential in importance might be due to the fact that BADDS captures a wider range of symptoms than ASRS, which only includes the 18 diagnostic criteria for ADHD. Another difference is that BADDS includes the inattentive symptoms of ADHD only, which are more common in adults. ASRS, by contrast, concerns both inattention and hyperactivity.

The present study concerned patients diagnosed with ADHD in adulthood only. Today, diagnosing adult ADHD is based on the assumption of a disorder emerging in childhood. Indeed, in order to meet the diagnostic criteria for ADHD, symptoms need to be present before the age of 12 (DSM 5; American Psychiatric Association, 2013). According to Moffitt, Houts, Asherson *et al*. (2015), in research, there is an ongoing discussion about the possibility of a late‐onset ADHD with its onset in adulthood, besides the typical childhood onset ADHD (Kooij *et al*., 2019). However, this assumption remains untested because there are as yet no longitudinal studies of the childhoods of individuals diagnosed with ADHD in adulthood. Thus, this issue remains unsettled and should be the focus of further research.

As such, is it important to examine progression of symptoms in adulthood. According to a meta‐analysis by Faraone *et al*. (2006) adult ADHD is more common than usually believed, especially with regard to patients with subclinical levels of symptoms (e.g., patients not fulfilling all criteria for the ADHD diagnosis according to the diagnostic manuals). The majority of adults with ADHD continue to struggle with substantial functional deficits related to their ADHD symptoms, especially when the ADHD‐diagnosis is combined with executive dysfunctions (Mattfield *et al*., 2014), and even in the subsyndromatic cases (Uchida, Spencer, Faraone & Biederman, 2018).

Concerning global functioning, we found at the group level that clinician‐rated functional impairment (GAF) scores remained unchanged over the course of 5 years. Being ≥ 60 on average, the results also indicate that these patients had relatively mild symptoms and experienced relatively minor impairments in daily living. Yet, more fine‐grained analyses showed that patients with the lowest functioning scores at baseline had the largest improvement at follow‐up. As noted by Brod, Pohlman, Lasser, and Hodgkins (2012), a lifetime of ADHD accumulates a number of functional problems that may be hard to correct even if the ADHD symptoms get milder or change in character with age. Most ADHD treatments are oriented towards targeting core symptoms; our findings suggest that treatments also need to be focused on increasing daily functioning for ADHD patients.

A clinically important question is whether there are predictors of long‐term outcome in adult ADHD patients. According to our OPLS models, high self‐reported levels of symptoms/functioning on a given scale at baseline predicted high levels on the same scale at follow‐up; no other factor was of importance when predicting outcome using self‐reported ADHD symptoms (BADDS and ASRS). However, the clinician‐rated functional impairment scores (GAF) at baseline, were the strongest predictors of the 5‐year score on the clinician‐rated symptomatic impairment score (CGI‐S). GAF baseline scores along with the sick leave factor also predicted the GAF improvement score. Because sick leave status is one of the indicators of global functioning, this result was not unexpected. Our results are in line with previous studies on this topic (Biederman *et al*., 2011; Karam *et al*., 2015; Lara *et al*., 2009; Lensing *et al*., 2013), and improvement in self‐rated ADHD was expected.

Five factors may account for the fact that the self‐rated ADHD symptoms improved (measured with ASRS and BADDS) whereas the overall level of clinician‐rated functioning (GAF) did not. First, many ADHD patients continue to be symptomatic as adults, but fewer continue to meet full diagnostic criteria (For example Karam *et al*., 2015). Second, we do not know to which extent patients adhered to treatment in present sample; it is known that compliant patients fare better than those who are not (Bejerot *et al*., 2010; Edvinsson & Ekselius, 2017; Lensing *et al*., 2013), even though the improvement might not reach the extent of normalization or reach levels of healthy controls (Shaw *et al*., 2012). Third, the GAFs in present sample was at baseline rated as ‘quite well functioning’ with only mild functional difficulties on average. Thus, the present sample was already at an adequate functioning level at baseline. Fourth, there are often clinically relevant symptoms accompanying ADHD that are not included as diagnostic criteria, such as sleep problems, executive dysfunction, or mood‐swings (Asherson *et al*., 2016). These problems may impact on overall functioning and life quality. Fifth, the fact that the patient rated ADHD symptoms and the clinician rated GAFs might be important: it is difficult for someone with lifelong ADHD to compare his or her own situation to that of someone without ADHD (Kooij, 2010).

Three potential predictors were conspicuous by their lack of importance for 5‐year outcomes. First, the retrospective WURS ratings of childhood ADHD symptoms turned out non‐significant. An earlier Swedish study reported clear links between high WURS scores and current ADHD symptoms in elderly people (Guldberg‐Kjär, Sehlin & Johansson, 2013). However, in that study comparisons were made between extremes: from a sample of almost 1,600 WURS ratings, the 30 lowest‐scorers were compared to the 30 highest‐scorers. In the present study, we attempted to relate WURS scores to current symptoms and functioning within a group of well‐defined ADHD patients. This proved unsuccessful, perhaps due to the difficulty in recalling symptoms from long ago, or due to the fact that this sample was diagnosed in adulthood and might not have had explicit ADHD‐difficulties as children. For example, Agnew‐Blais, Polanczyk, Danese, Wertz, Moffitt and Arsenault (2016) found an adult onset ADHD prevalence of 5.5% in their UK cohort, and that 67.5% of their sample of adults diagnosed with ADHD would not have met diagnostic criteria for ADHD as children. Their group of 112 patients showed lower levels of externalizing problems and higher IQ in childhood compared to the group of persistent childhood ADHD, results comparable to present findings.

Second, presence of comorbid psychiatric problems was not associated with outcome. This is surprising given that many other studies show that psychiatric comorbidity worsens the prognosis of ADHD (Roy *et al*., 2016). Comorbidity is clinically important and a factor contributing to both persistence in adulthood (Faraone *et al*., 2015; Kooij *et al*., 2019; Roy *et al*., 2016), and to finding the most effective treatment (Instanes, Haavik & Halmoy, 2016; Kooij *et al*., 2019). We used only registered comorbid diagnoses, which excluded potential contribution from sub‐threshold comorbid psychiatric symptoms, a possible explanatory factor. Another possible reason for lack of impact from comorbid psychiatric diagnoses is the small sample and thereby less power to detect differences, since comorbid ADHD is more common than clean‐cut ADHD in clinical samples (77.1% prevalence of psychiatric lifetime comorbidity; Sobanski *et al*., 2007).

Third, ADHD medical treatment did not appear important for the 5‐year symptomatic or functional outcomes. According to the medical records, 35 (65.4%) patients were medicated at both baseline and at follow‐up. We have no data on how compliant these patients were, but for 22 of them the median number of months on medication was 48 (i.e., approximately 80% of the examined time span). The fact that this was a naturalistic study lowers the quality of the medical data, and Shaw *et al*. (2012) have described the poor systems for follow‐ups and difficulties in maintaining long‐term medical administration in the healthcare systems as examples of contributors to the sparse number of existing long‐term, naturalistic follow‐ups in ADHD (Shaw *et al*., 2012). Even so, the absence of effect on 5‐year outcome is notable since medication is considered first‐line treatment in adult ADHD (Kooij *et al*., 2019), and multiple studies confirm the value of ADHD‐medication in terms of alleviating core symptoms (Kooij *et al*., 2019). However, as remarked by Cortese *et al*. (2018) these positive outcome studies are mostly short‐term and seldom longer than 12 weeks. Likewise, naturalistic studies on children and adolescents reveal beneficial effects of ADHD medications in the short‐term but not in the long‐term (Asherson, Chen, Craddock & Taylor, 2007; Jensen, Arnold, Swanson *et al*., 2007; Molina, Hinshaw, Swanson *et al*., 2009; Nylander, 2018; Storebø, Ramstad, Krogh *et al*., 2015; The MTA Cooperative Group, 1999, 2004; van Lieshout, Luman, Twisk *et al*., 2016). Thus, the present 5‐year study provides yet another example of a possible failure to detect favourable effects of ADHD‐medication in the long run. This failure might reflect a true dissipation of the symptom‐reducing effects of the drug, but complementary possibilities includes issues related to drug discontinuation (Zetterqvist, Asherson, Halldner, Långström & Larsson, 2013), and to adherence‐to–medication (Bejerot *et al*., 2010). As emphasized by Cortese *et al*. (2018), there is an urgent need for assessing the long‐term effectiveness of ADHD medications to refine treatment choices and clinical management for adult ADHD patients (Arnett & Stein, 2018; Asherson *et al*., 2016).

### Limitations

First and most important, the lack of detailed information about type of central stimulants, dosage, individual stop/start‐patterns or co‐medication might weaken the conclusions reached in the present study. This is a common difficulty in naturalistic, uncontrolled studies of real‐life patients in real‐life settings where the information rests upon the patient’s compliance and self‐reporting skills. Second, the relatively small sample size might have impeded our ability to detect somewhat less powerful predictors. Third, in the present study the number of patients reporting comorbid substance abuse was surprisingly low, as were the overall rate of comorbidity, which might indicate that the present sample was not entirely representative in all aspects (Capusan, Bendtsen, Marteinsdottir & Larsson, 2019). The primary advantage of the present study is its time length and its high ecological validity. Another advantage is the use of statistics designed to handle multicollinear datasets with few observations relative to the number of variables.

## CONCLUSIONS

Patients diagnosed with ADHD in adulthood showed a decrease in ADHD symptom burden over the course of 5 years according to both self‐reports and clinicians’ judgements. However, at case closure the ADHD patients, as a group, remained impaired compared to controls. Medication, comorbidity, IQ, age and sex are all factors known to predict short‐term outcomes, but did not anticipate the long‐term outcomes in the present study.

This research was supported by grants from the Swedish Research Council (2018‐02653) and the Swedish Federal Government under the LUA/ALF agreement (ALF 20170019 and ALFGBG‐716801).

We are deeply grateful for the participation of all patients contributing to this research, and we are indebted to the collection team at the Northern Stockholm Psychiatry Clinic that worked to recruit them. We especially wish to thank study nurse Lena Lundberg and data manager Mathias Kardell. ML declares that, over the past 36 months, he has received lecture honoraria from Lundbeck pharmaceutical. No other equity ownership, profit‐sharing agreements, royalties, or patent. The other authors have no competing interests to declare. The datasets generated and/or analyzed for the current study are not publicly available due to the Swedish law for register data. But data will be available from the corresponding author on reasonable request.

## Data Availability

The datasets generated and/or analysed for the current study are not publicly available due to the Swedish law for register data. But data will be available from the corresponding author on reasonable request.
